# Frequent attenders in general practice: problem solving treatment provided by nurses [ISRCTN51021015]

**DOI:** 10.1186/1471-2296-6-42

**Published:** 2005-10-12

**Authors:** B Schreuders, P van Oppen, HWJ van Marwijk, JH Smit, WAB Stalman

**Affiliations:** 1 Department of General Practice, VU University Medical Center, Amsterdam, the Netherlands; 2 Department of Psychiatry, VU University Medical Center, Amsterdam, the Netherlands; 3 Institute for Research in Extramural Medicine, VU University Medical Center, Amsterdam, the Netherlands

## Abstract

**Background:**

There is a need for assistance from primary care mental health workers in general practice in the Netherlands. General practitioners (GPs) experience an overload of frequent attenders suffering from psychological problems. Problem Solving Treatment (PST) is a brief psychological treatment tailored for use in a primary care setting. PST is provided by nurses, and earlier research has shown that it is a treatment at least as effective as usual care. However, research outcomes are not totally satisfying. This protocol describes a randomized clinical trial on the effectiveness of PST provided by nurses for patients in general practice. The results of this study, which currently being carried out, will be presented as soon as they are available.

**Methods/design:**

This study protocol describes the design of a randomized controlled trial to investigate the effectiveness and cost-effectiveness of PST and usual care compared to usual care only.

Patients, 18 years and older, who present psychological problems and are frequent attenders in general practice are recruited by the research assistant. The participants receive questionnaires at baseline, after the intervention, and again after 3 months and 9 months. Primary outcome is the reduction of symptoms, and other outcomes measured are improvement in problem solving skills, psychological and physical well being, daily functioning, social support, coping styles, problem evaluation and health care utilization.

**Discussion:**

Our results may either confirm that PST in primary care is an effective way of dealing with emotional disorders and a promising addition to the primary care in the UK and USA, or may question this assumption. This trial will allow an evaluation of the effects of PST in practical circumstances and in a rather heterogeneous group of primary care patients. This study delivers scientific support for this use and therefore indications for optimal treatment and referral.

## Background

In primary care the prevalence of psychological problems (e.g. depression, anxiety, stress, somatization, unexplained or functional symptoms) ranges from 30% to 70%. Patients with these complaints, symptoms or disorders frequently visit their general practitioner [1] and only 3% of all patients are referred to a specialist. This implies that mental health care is a core activity in primary care [2]. For many of these complaints and symptoms no evidence-based treatment is available [3]. There is a clear need for an effective treatment for common mental disorders in primary care.

### Problem Solving Treatment(PST) in primary care

**Figure 1:**
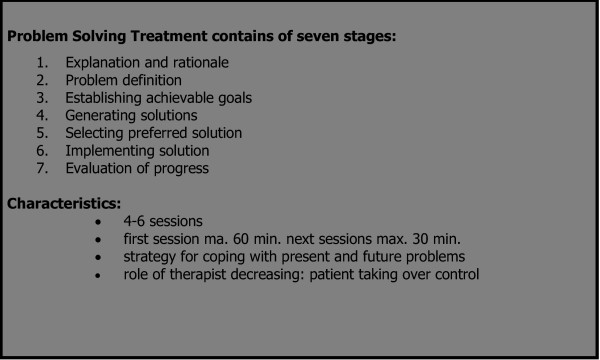
The seven stages of problem solving treatment.

In 1971 D'Zurilla and Goldfried published a theory in which problem-solving was defined as a cyclic process in five stages: problem orientation; problem definition; generation of alternative solutions; decision making, and solution implementation and was called problem solving therapy [4]. Since then, problem solving therapy has been applied for a wide range of psychological problems in all kinds of areas. In 1995 Gath and Mynors-Wallis conducted an experiment based on a basic form of PST in primary care. This is strictly protocollized and based on the principles of cognitive behavioral therapy (CBT) [5]. The treatment is brief and focuses on practical skill-building. It consists of a maximum of six sessions, each of which contains seven steps of problem-solving (see Figure 1) which are applied in a systematic manner towards problem resolution. The rationale is that it increases the patient's understanding of the relationship between everyday problems and psychological symptoms. The goal of PST is to help patients to regain control of their lives.

There is evidence that PST can be an effective way of helping patients, and in particular patients with depression, to deal with psychological problems. One earlier study showed the superiority of PST over placebo but no superiority over amitryptiline [6]. A second study showed equal results in clinical outcomes between patients who received PST and patients who received usual care from their GP [6]. When community nurses provided PST the results were the same as for usual care from the GP, but the economic evaluation was more positive for the PST group [7]. Patients with minor depression who received PST showed the same improvement as patients who received a placebo, but their symptoms improved somewhat more rapidly than those of patients who received a placebo during the latter treatment. Patients with dysthymia who received PST and paroxetine showed significantly more improvement than patients who received a placebo [8]. Compared to other GP interventions there is good evidence PST is effective for major depression [1].

### PST provided by nurses as a potential option

Patients with psychological problems need more time than is available in general practice. The usual 10-minute consultation with a GP is generally too short to explain and explore these psychological problems. To complicate matters more, these problems are often interwoven with physical issues such as fatigue and sleeplessness. Furthermore, patients are ambiguous in presenting their symptoms [9]. Given this fact, in combination with the high prevalence of psychological problems in primary care, treating these patients will result in a shift of tasks to nurses. Especially nurses who are skilled in working with psychiatric patients, may become indispensable in primary care [10]. Nurses can be successfully trained in the techniques of PST and can provide effective PST for primary care patients [11]. Recent results show that a CBT protocol for panic-disorder can adhered by a therapist with minimal of or no CBT experience [12].

There are several issues which stimulate further investigation. First, PST may be the way forward in the Netherlands, where GPs have a heavy workload and patients need better tailored collaborative forms of care, focused on self-help and education.

There are still very few nurses working in Dutch general practice and although preliminary experiments are taking place to enhance and define the role of nurses in primary care, PST could be a welcome innovation in their task profile. So, innovative projects in primary care in the Netherlands are needed.

Secondly, there is a lack of research outcomes on the effectiveness of talking treatment for anxiety symptoms in patients. In this trial, patients with depressive as well as anxiety symptoms will be included. Only one study has reported substantially better outcomes for primary care patients with panic disorder, who received CBT and pharmacotherapy from a therapist with minimal or no CBT experience, like a nurse, than patients with usual care only from their GP [12].

Third, PST in primary care could prevent or stimulate a referral to secondary care for patients with complaints which cannot be treated in primary care. This would also stimulate better tailored collaborative forms of care and prevent the deterioration of complaints.

The primary aim of the present trial is therefore to investigate whether PST for patients with psychological problems provided by nurses in primary care, is effective.

## Methods

### Design

A randomized, controlled trial is being carried out to evaluate the effects of PST. At least 160 primary care patients will be included; 80 will receive usual care and PST and 80 will receive usual care only. At baseline, after the intervention and after 3 and 9 months the patients will be asked to fill in a questionnaire, and at baseline and after 9 months they will be asked to cooperate in a (diagnostic) telephonic interview. Primary outcome is the reduction of symptoms, and other outcomes measured are improvement in problem solving skills, psychological and physical well being, daily functioning, social support, coping styles, problem evaluation and health care utilization.

The Medical Ethics Committee of the VU Medical Center in Amsterdam approved the study design.

### Study population

The study population will consist of Dutch-speaking adults (18+) who visited a participating GP more than three times in the last six months. To asses whether psychological problems are present, the General Health Questionnaire 12 item version (GHQ-12) will be used for screening [13]. At random we visited the participating GP practices to ask patients to fill in our screening questionnaire while they where waiting to see their GP. If they had a score negative score on more then three out of twelve questions (indicating the presence of psychological problems) and if they were willing to participate, they were included. Patients were excluded from the study if they: received any treatment in mental health care; suffered from a severe (psychical) disease or personality-disorder; accepted no other explanation for their complaints than a somatic rationale; and patients with an non-consistent medication for anxiety or depression. Patients with severe drug addictions, suicidal wishes or mental retardation were excluded. An external researcher conducted block-randomization, so the allocation was concealed.

#### Intervention: usual care and PST provided by nurses

Consistent with earlier research on PST training skills [11], the nurses in this RCT were trained for two days by experienced supervisors who were also members of the original Oxford research group[6]. The nurses were closely supervised by means of video and audiotapes. A CBT supervisor will carry out supervision after the training, for one hour every three weeks. The nurses will deliver audiotapes and PST protocol forms to the supervisor. Consistent with advice in earlier research [11] before the nurses started treating patients in the trial, they treated four patients to practice their problem-solving skills after the training. The patients are also seen by their GP for general health management if necessary.

#### Usual care: health management provided by the GP

The consult is intended to be as natural as possible so that the GP will not influence the quality of the usual care provided. Many GPs use the guidelines issued by the Dutch College of General Practitioners [14]. The guidelines for psychological complaints such as anxiety and depression describe management options as (anti-anxiety of anti-depressant) medication and/or 'watchful waiting' if a referral seems unnecessary [15].

### Outcome measures

#### Primary outcome: reduction of symptoms measured with the HADS

The Hospital Anxiety and Depression Scale (HADS, [16]) is used to monitor symptom levels of anxiety and depression in the study population. The questionnaire consists of 14 items to which answers can be given on a 4-point scale (0–3). The HADS is considered to be unbiased by coexisting general medical conditions, and changes in HADS scores can therefore be used to calculate an objective effect size of the treatment provided (calculations described in 'Sample size'). In the Dutch validation of the HADS [16] the primary care patients have a mean of 6.2 (SD 3.8)for anxiety and 3.7(SD 3.4) for depression with a total mean of 9.9 (SD 6.1). Reliability for these patients is a Cronbach's alpha of .82 for the total score. The HADS is found to perform well in assessing the severity of symptoms [17].

#### Secondary outcomes: reduction of symptoms measured with the PHQ

The Patient Health Questionnaire (PHQ) is designed to facilitate the diagnosis of common mental disorders in primary care patients [18]. The PHQ is a self report version of the Primary Care Evaluation of Mental Disorders (PRIME-MD). The questions do not only focus on mood disorders but also about functional impairments, life stressors and specific events (such as menstruation, pregnancy and childbirth). Its diagnostic validity is good, and patients feel comfortable filling in the questionnaire [19]. There is a 15-item questionnaire for men and a 16-item questionnaire for women, and the scoring range varies. We consider that a decrease in the score on this questionnaire after the intervention represents a reduction in mood disorders, functional impairments, life stressors and distress about specific events.

#### Improvement in problem-solving skills

The Social Problem Solving Skills-Revised (SPSI-R, 15) is a 52-item, self-report inventory, which is designed by D'Zurilla to measure problem-solving skills [20]. The SPSI-R consists of five factors: 1) positive problem orientation (PPO), 2) negative problem orientation(NPO), 3) rational problem solving (RPS), 4) impulsivity/carelessness style (ICS) and, 5) avoidance style(AS). Alphas for these five scales range from .76 to .92 [21] an test-retest reliability ranges from .72 to .88 [20].

#### Psychological and physical well-being

This will be measured with the Short Form-36 (SF-36) which contains 36 questions and standardized response options and relating to eight different areas (multi-item): physical functioning, role limitations due to psychical health problems, bodily pain, general health perceptions, vitality, social functioning, role limitations due tot emotional problems, and general mental health [22]. The mean alpha for reliability in the general population is good, as well as validity which makes the SF-36 a practical instrument for use in the general population.

#### Social support

The Social Support Inventory is a questionnaire which comprises 20 descriptions of social support pertaining to emotional support, informative support, social companionship, or instrumental support. Together these items give an overall representation of satisfaction with social support (the perceived adequacy). It is a reliable and brief measurement instrument which is not influenced by psychological distress [23].

#### Coping-styles

The Ways of Coping Questionnaire (WAYS) (the Dutch adaption is called the VOMS: Vragenlijst over Omgaan met Situaties) is based on the Lazarus and Folkman transactional coping theory of [18]. It measures coping processes, not coping dispositions or styles. The WAYS can assess and identify thoughts and actions that individuals use to cope with stressful encounters in everyday life. The WAYS measures eight coping factors: confrontive coping, distancing, self-controlling, seeking social support, accepting responsibility escape-avoidance, planful problem solving, and positive reappraisal.

##### Rumination

Actual scientific reports suggest that rumination is a significant, and probably prognostic, factor for depression. The rumination scale (RRS) measures the extend to which people ruminate [24]. Rumination is seen as a coping style and characterizes depressive mood. The reliability is good and its validity is satisfying [25,26].

#### Problem evaluation

This will be assessed with a brief, qualitative questionnaire about medical outcomes, the care, the illness and the treatment of the patients, as experienced by the patients, to complement all the quantitative questionnaires. We chose the PSYCHLOPS(also known in the literature as MYMOP [27,28]) to evaluate the problems patients experience and the progress they make over time.

#### Health care utilization

The Trimbos/iMTA questionnaire for Costs associated with Psychiatric Illness (Tic-P) is used to measure the amount of health care received by the patients and to register sickness absence from work [29] Furthermore, we chose the EQ-5D (or Euroqol, [30]) because this is a standardized measurement instrument for a wide range of health conditions which provides a simple descriptive profile and a single index value for health status. The EQ-5D (or EuroQol) was originally designed to complement other instruments such as the SF-36, and it is administered to assess a patient's general health status, in 5 dimensions: mobility, self-care, usual activities, pain/discomfort and anxiety/depression[1]. Because each of the five dimensions can be sub-divided into 3 levels a total of 243 health states can be assessed. Using the Dolan model (1997) the total score will be expressed in utilities [31]. The official Dutch translation of the Euroqol will be administered [31,32]. Incremental cost-effectiveness ratios will be calculated, in which the difference in costs between intervention subjects and control subjects will be divided by the difference in effects between both groups. Incremental cost-utility ratios will also be calculated in which the difference in costs between the two groups will be divided by the difference in QALYs gained between the two groups.

### Power/analysis

Randomization takes place at patient level. To evaluate the effects of the randomization, descriptive statistics will be used to compare the baseline measurements of the two groups. If necessary, differences between baseline variables on relevant characteristics (such as baseline HADS score) will be entered as covariates in the analysis.

To detect a clinically relevant difference between interventions (effect size of 0.4) with the primary outcome measure HADS (power of .90 and an alpha of .05) 130 completers are needed. In both conditions there will be 65 completers. We estimate the drop-out rate to be on 20%, so we therefore need 160 participants. If 20 practices cooperate, we will need to include 8 patients in every practice. We expect a non-response of 50%, so we will need to screen 16 patients in every practice. In a sample of patients with mixed symptoms of anxiety and depression, Cropper et al. [33]observed a mean overall HADS score of 6.25 at baseline. In the Dutch validation [16] the primary care patients have a mean on anxiety of 6.2 (SD 3.8) and 3.7(SD 3.4) for depression with a total mean of 9.9 (SD6.1). We consider a standardized mean difference (SMD) of 0.4 (p = 0,05) on the primary outcome (HADS) to represent a relevant improvement in the PST group versus the usual care group[34].

#### Analysis

Linear regression models will be used to examine differences in investigate on the HADS. Scores will be entered in a repeated measure design (GLM), and (optional) covariates will be differences at baseline level. Repeated measures with several independent variables will be used to investigate differences in improvement in all secondary outcome measures between groups. The analyses will be performed on a per protocol basis ('completers'), as well as according to the 'intention-to-treat' principle. Trend analyses will also allow 'last observation carried forward' analyses. To assess whether protocol deviations have caused bias, the results of the intention-to-treat analyses will be compared with analyses of the PST group, including the completers.

### Sample size

A GP in the Netherlands has an average of 80 consultations a week (children excluded). Six of the patients who consult their GP had done so more than three times in the previous six months and were 'frequent attenders'. With a response of 50%, three patients a week per general practice will be sufficient, but: only one third of them will meet the inclusion criteria. This implies that 1 patient can be included per practice per week. To include 160 patients with a screening in two practices per week, will take approximately one and a half years.

#### Arguments for publishing a design

The primary goal of presenting the design of this study before the results are available is to offer the reader the opportunity to consider the methodological quality of this study more critically and this is also a benefit for caregivers, because this extensive information provides more insight into the practical applications of the study intervention.

Publication also prevents publication-and-analysis bias. Trials that lead to adverse or negative results are less likely to be submitted for publication [35]. This can be avoided by publishing a priori the design of a study and the plans for analysis. Not only will the researchers be more inclined to publish the results, but transparency will also be increased and, in any case, data can be requested from the researcher for inclusion in a systematic review.

## Discussion

Our results may either confirm that PST in primary care is an effective way of dealing with emotional disorders and a promising addition to the primary care in the UK and USA, or may question this assumption. This trial will allow an evaluation of the effects of PST in practical circumstances and in a rather heterogeneous group of primary care patients.

### Strengths and limitations

Many methodological requirements for a high quality trial are met [36]. Allocation is concealed through block-randomization by an external researcher. Recruitment of responders will be in the GP's waiting room, without the GP knowing where and how randomization will take place. The methodology used in this trial will overcome concerns of selection bias. The relevance to the Dutch primary care seems sufficient and the generalisability of our sample to patients in everyday practice seems high. The sample size is large, when compared to other trials on psychological problems in general practice [37]. Additionally, whether the outcome is negative or positive, the project will give a clearer understanding of who might or might not benefit from this treatment.

Another strength of this study is the chosen primary outcome, the self-report HADS. The HADS is a well-known questionnaire to measure reduction of symptoms of anxiety and depression. Many previous trials in psychiatry have relied on assessments of the therapist. Another strength is the combination of quantitative assessments and a qualitative process evaluation. If the psychiatric symptoms decline more in the intervention group than in the usual care group, we can therefore safely attribute this to PST treatment. If not, we will explore mechanisms This will provide useful information about implementing the intervention. A practical strength of the study is that the intervention can take place without disturbing care as usual, both in the study as in future implementation.

Some limitations of the present study need to be addressed. It is possible that patients are less motivated to attend PST treatment since screening of all participants takes place in the waiting room of the GP's. This may be lead to a higher dropout percentage in the PST-condition in comparison with the dropout percentage of the CAU.

This study may be criticized because of the lack of a control group without treatment. However, patients are always free to visit their general practitioners. Furthermore, naturalistic studies of the longitudinal course of anxiety and depression indicate that patients who have these symptoms longer than three months are suffering from chronic symptoms [38,39].

Other potential criticism concerns the suitability of PST for primary care. Although PST seems suitable for primary care, provision of PST by trained nurses will not always be available in daily practice of small primary care practices.

## Conclusion

PST is a short psychological treatment for use in general practice. This study delivers scientific support for this use and therefore indications for optimal treatment and referral. Study completion is anticipated for January 2006, with results available in May 2006.

## Competing interests

The author(s) declare they have no competing interests.

## Authors' contributions

PvO and HvM developed the design of the randomized clinical trial and participated in writing the article. WS and JS advised on the content of the article. BS conducts the research and wrote the article. All authors provided comments on the drafts and have read and approved the final version.

## Pre-publication history

The pre-publication history for this paper can be accessed here:


